# A qualitative assessment of faculty perspectives of small group teaching experience in Iraq

**DOI:** 10.1186/s12909-015-0304-7

**Published:** 2015-02-15

**Authors:** Abubakir M Saleh, Nazar P Shabila, Ali A Dabbagh, Namir G Al-Tawil, Tariq S Al-Hadithi

**Affiliations:** 1 Department of Community Medicine, College of Medicine, Hawler Medical University, Erbil, Iraq; 2 Department of Surgery, College of Medicine, Hawler Medical University, Erbil, Iraq

## Abstract

**Background:**

Although medical colleges in Iraq started recently to increasingly use small group teaching approach, there is limited research on the challenges, opportunities and needs of small group teaching in Iraq particularly in Kurdistan Region. Therefore, this study was aimed to assess the small group teaching experience in the 4^th^ and 5^th^ year of study in Hawler College of Medicine with a focus on characterizing the impressions of faculty members about how small group teaching is proceeding in the college.

**Methods:**

A qualitative study based on semi-structured interviews with 20 purposively selected faculty members was conducted. An interview guide was used for data collection that was around different issues related to small group teaching in medical education including planning, preparation, positive aspects, problems facing its implementation, factors related to it and recommendations for improvement. Qualitative data analysis comprised identifying themes that emerged from the review of transcribed interviews.

**Results:**

Participants reported some positive experience and a number of positive outcomes related to this experience including better controlling the class, enhancing students’ understanding of the subject, increasing interaction in the class, increasing the students’ confidence, enhancing more contact between teachers and students, improving the presentation skills of the students and improving the teacher performance. The participants emphasized poor preparation and planning for application of this system and highlighted a number of problems and challenges facing this experience particularly in terms of poor infrastructure and teaching facilities, poor orientation of students and teachers, inadequate course time for some subjects and shortage of faculty members in a number of departments. The main suggestions to improve this experience included improving the infrastructure and teaching facilities, using more interactive teaching methods and better organization and management of the system.

**Conclusions:**

Despite what the faculty perceived as the college’s failure to provide physical settings or training for small group learning to the faculty and the students, the faculty members were able to articulate positive experiences and outcomes associated with their college’s efforts to introduce teaching in smaller group sessions.

**Electronic supplementary material:**

The online version of this article (doi:10.1186/s12909-015-0304-7) contains supplementary material, which is available to authorized users.

## Background

The practice of medicine in the 21^st^ century will require multidisciplinary and collaborative medicine [1]. Undergraduate medical education needs ongoing improvements to meet the changing demands of medical practice in the 21^st^ century. The main direction of change is to shift more from teacher to student because it is more aligned with student centered learning, teaching is adaptable to meet the needs of every student, and students taught in this way retain more material for longer periods of time [2]. This approach prepares the learners to be independent and self-reliant in their learning, efficient and more responsive to the needs of the fast-changing and ever-demanding field of medicine [3].

Small group teaching is indicative of the movement from a traditional teacher-centered approach to a more student centered learning approach and it is characterized by student participation and student-teacher interaction [4]. It can give the students the chance to monitor their own learning [5,6]. Among the educational objectives that can be achieved through the use of small group teaching methods are the development of higher-level intellectual skills such as reasoning and problem-solving and acquisition of interpersonal skills such as listening, speaking, arguing, and group leadership. These skills are also important to medical students who will eventually become involved professionally with patients, other health care professionals, community groups, learned societies and the like [7]. Small group teaching is one of the most difficult and highly skilled teaching techniques in medical education and should be planned carefully and both the students and tutor should know how to work with it [8].

Medical education in Iraqi medical schools is based on the 6-year Edinburgh traditional curriculum [9]. The first three years of study involve basic medical sciences while study of clinical sciences starts in the third year and is mainly focused in the 4^th^ and 5^th^ years of study. Teaching in the 4^th^ and 5^th^ years of study involves both theoretical and practical sessions in the different clinical topics. The 6^th^ year of study is clinical training in the different clinical departments at hospitals. The final year is devoted for practical clinical training. After completion of the study, students will be awarded MBChB degree that will make them eligible to be registered with the Doctors’ Syndicate and then able to practice medicine. A two year residency rotating internship will usually follow their registration. Medical study and training in Iraq is entirely in English language and is free.

According to a self-study report that has been prepared for the National Accreditation Committee of Iraqi Colleges of Medicine, Hawler College of Medicine has implemented a number of initiatives to redesign the delivery of courses to enable a shift from traditional teaching approaches to student centered learning. One of these initiatives involved dividing the students into smaller groups to achieve more interactive teaching. It was thought that having a smaller number of students in the theoretical and practical sessions will encourage more interactive teaching and the teaching time will be more adequate to ensure students’ participation in discussions. This process has started in 2007 for 5^th^ year students and then extended to 4^th^ year students. The students of around 160 were divided into smaller groups in theoretical sessions and each group take theoretical and practical sessions consecutively where one subject will be taken over a specific period of time. A small group is typically between 30 to 40 students for theoretical sessions and between 12 to 15 students for practical sessions. In general, most of the small group sessions still depend on lectures. In the previous didactic large group system, students were taking all subjects concurrently together according to specific schedule [10].

As no research has assessed this newly introduced small group teaching experience in Hawler College of Medicine, this study was aimed to assess the small group teaching experience in the 4^th^ and 5^th^ year of study in Hawler College of Medicine with a focus on characterizing the impressions of faculty members about how small group teaching is proceeding in the college.

## Methods

### Design/setting

This qualitative study based on semi-structured interviews with a sample of faculty members was carried out in Hawler College of Medicine, Erbil city, Iraqi Kurdistan Region between October, 2011 and October, 2012.

Hawler College of Medicine is situated in Erbil city, the capital of Kurdistan Region. The college was established in 1977 as part of the University of Suleymania in Suleymania governorate, and then moved to Erbil governorate in 1982 as one of the University of Salahaddin’s colleges till 2005. Then, Hawler Medical University was established in Erbil and the College of Medicine became one of its main institutions. It is governed by the college council headed by the dean [10].

### Participants

Twenty faculty members were purposively selected based on their knowledge and involvement in the experiences of small group teaching in the college, and ability to answer the research questions. The operational definition of what constitutes a small group in Hawler College of Medicine context and experience includes having between 30 to 40 students for theoretical sessions and between 12 to 15 students for practical sessions. The study participants included five heads of departments, seven coordinators of departments, one member of quality assurance committee, one member of accreditation committee and six faculty members with special interest and experience in medical education. The study participants were accessed through the Dean of the college who acted as the gatekeeper for the study. These participants were invited to the interview through personal meetings.

At the beginning of the interview, the participant information sheet was delivered with a written consent form. It was made clear that participation is voluntary and participants can withdraw from the study at any time without any consequences. The participants’ consent was obtained before starting the interviews. The study was approved by the Research Ethics Committee of Hawler College of Medicine.

Three pilot interviews were held in October and November, 2011 with three faculty members in the college. These interviews were not included in the study. The main aim of the pilot interviews was to test the clarity and relevance of the questions and to give the opportunity to the researcher to practice and become familiar with face-to–face interview. The pilot interviews also helped to assess the type of data that would emerge from the interviews.

### Data collection

A semi structured interview was used in this research. A list of open-ended questions was prepared. Although all areas were covered, the order and the actual wording of these questions were different for different respondents. Probes were used for clarification of some questions, and at times some extra questions have been added in for some respondents depending on their answers for the prepared list of questions.

The interview guide was developed around different issues related to small group teaching in medical education including planning, preparation, positive aspects, problems facing its implementation, factors related to it and recommendations for improvement. The interview guide was composed of eight sections (Additional file 1). The interview protocol asked the faculty to characterize their impressions about how small group teaching was proceeding in the institution, rather than focusing on the individual instructors to report on their own direct efforts in implementing it.

All interviews were held in Kurdish language except two interviews conducted in Arabic. The interviewer‘s mother tongue is Kurdish and he is fluent in Arabic language so there was no need for translators in the interviews. One of the basic requirements for any qualitative work, including interview, is the fluency in the cultural language of the interview [11].

The interviews were recorded, using a digital voice recording device, to allow detailed analysis of the content of the interview. The participants’ consent for recording the interviews was obtained. Only a few hand written notes were made during the interviews as the researcher focused on maintaining interaction with the respondents during the interviews through direct eye contact. The sample size was adequate to ensure achieving saturation as no new concepts were produced in final interviews.

The average duration for each interview was almost one hour, and the bulk of the interviews were transcribed by using Microsoft Word Software and translated fully into the English language with the exception of some long responses that were summarized.

### Data analysis

Qualitative data analysis comprised identifying themes that emerged from the review of transcribed interviews with study participants. Given the manageable length of 20 interviews, no software was used to analyze the data.

The following steps have been taken for analyzing the data of the study:

Step 1: Short notes have been made about important topics in each interview during interviewing the respondents.

Step 2: Transcription (with translation) of all the interviews.

Steps 3: Two authors reviewed the transcripts independently and identified many topics to describe all contents of data. Then these topics were categorized into different topics “open coding”. The two authors compared and discussed the generated codes and themes and reconciled the differences.

Step 4: Similar topics were put together and this reduced the number of topics.

Step 5: Numerical codes were given to these different topics.

Step 6: Topics with the same code in all interviews were put together in a separate supplementary file using a word processor program.

Step 7: These organized data have been used for writing up the results of the study.

The emerging themes were organized under eight general categories: planning and preparation; positive aspects; problems and challenges; difference in students’ behavior in small group teaching and large group teaching; teaching methods in small group; size of group; teacher-student relationship in small group; and recommendations for improvement.

## Results

### Demographic and professional characteristics

Of 20 participants, 16 were male, eight were in the age group 45–49 years, 11 were lecturers in different clinical departments and seven had 15–19 years of teaching experience (Table 1).

**Table 1:** **Sex, age and professional characteristics of study participants**

Characteristic	No.	(%)
*Sex*		
Male	16	(80)
Female	4	(20)
*Age group*		
40-44	3	(15)
45-49	8	(40)
50-54	4	(20)
55-59	2	(10)
≥60	3	(15)
*Academic title*		
Professor	3	(15)
Assistant professor	4	(20)
Lecturer	11	(55)
Assistant lecturer	2	(10)
*Teaching experience (years)*		
5-9	6	(30)
10-14	5	(25)
15-19	7	(35)
≥20	2	(10)

### Planning and preparation

The participants emphasized the need for adequate planning and preparation for implementing the new experience that should include preparation of infrastructure, faculty members and students. One of the participants mentioned that there were no preparations before implementing this experience at all levels:

*“I do not think that there were any preparations. I think there should be planning for at least two to three years, preparation of all requirements then implementing as a pilot in a one year of study to see defects and problems. After that it should be implemented on a wider scale”.*

Some of the participants agreed that there was some preparation regarding teaching halls and facilities both in the college and hospitals:

*“Some halls were prepared in the university with teaching facilities which I think could cover 80% of the requirements for small group teaching”.*

One participant mentioned that they were lucky because the manager of the hospital was very cooperative with them and prepared everything for them:

*“We were lucky because I raised this issue to the attention of the manager of the maternity hospital and she was very cooperative and allocated one entire unit of the hospital for teaching. This unit contains many rooms equipped with teaching facilities, so we had very good space for teaching, not like other departments which had problems with limited space”.*

Regarding faculty members’ preparation, majority of them agreed that there was no preparation and even most of them did not know what was going on:

*“For me there were no proper preparations. Other faculty members were also not prepared. Majority of them did not know what was going on. I can say that more than 95% of faculty members were not aware about the new situation”.*

One participant mentioned that there was a course for developing student-centered learning in the university in coordination with the University of Glasgow from United Kingdom:

*“For faculty members, there was a course for developing student-centered learning in the university in coordination with University of Glasgow and I was one of them”.*

Another participant commented on this course and mentioned that it was limited to few faculty staff in different departments in the college:

*“What I have observed in student centered learning training workshop, we were only 20 faculty members and we have more than 230 faculty members in the college. So if we calculate the ratio, it will be less than 10% of faculty members that are well trained about student centered learning in general including management of small group teaching ”.*

Majority of the participants agreed that students were not prepared well for this change in their curriculum:

*“Students were not prepared for this new situation and they were not aware about new teaching methods”.*

Most of them agreed that in the beginning of each course they do some orientation for the students regarding the schedule and the timetable of the course:

*“In the first day of the course, we give instructions and information about the schedule and the timetable of the course”.*

One participant mentioned that in the beginning of the implementation of this experience in 5^th^ year study, students resisted it and they went to the street for demonstration against it:

*“Students were struggling and they went to the street for demonstration against this decision because they felt that they have lost some advantages of the large group teaching especially for those who were not interested and were not paying much attention to the classes”.*

### Positive aspects

The participants articulated positive experience and outcomes associated with this new experience including better controlling the class, enhancing students’ understanding of the subject, increasing interaction in the class, increasing the students’ confidence, enhancing more contact between teachers and students, improving the presentation skills of the students and improving the teacher performance:

*“Teachers can better control the class and attract the attention of the students during the class”.*

*“Students are more comfortable for asking questions and taking part in discussions whether they understand the subject or not”.*

*“Students now understand the subject more because there are few students with the teacher and more discussion and this will make students understand the subject more”.*

*“Increasing the students’ confidence as they were unable to ask questions in front of 140 students while in small groups, students are more confident and not shy. This will increase their confidence and enhance their presentation skills especially if the relation between the students and the teacher was good”.*

*“Teachers know all the students in the group. Although the period of each course is short but I think it is enough to know all of them”.*

*“Teacher will learn new information because students read the subject before coming to the lecture. Students prepare themselves for the subject, so the teacher may learn some new information from the students”.*

### Challenges and problems

Most of the participants agreed that there are many problems regarding infrastructure such as lack of appropriate teaching halls for small group teaching, unavailability of spaces for faculty members in the college, inadequate library, and poor access to internet:

*“The main problem is the building of the college which is not suitable for teaching. We cannot do much positive changes because of the building. It is like broken window phenomena, even if one was interested to do something new but s/he will be disappointed and after a while s/he will join the old system and lose his motivation”.*

Many problems related to the faculty members were reported by the participants such as inadequate number of staff in some departments; staff not trained well for teaching small groups; staff dissatisfaction with the experience; lack of foreign faculty members and some teaching staff are not fully committed to the teaching in the college:

*“Inadequate number of faculty members in some departments of the college which make them under stress because they have to do extra work”.*

*“Some of the faculty members are not satisfied with this method of teaching and some of them are satisfied but they do not know how to teach in small group”.*

The participants mentioned many problems related to the students during implementation of this experience including large number of students in each group; inadequate orientation of students; mentality of students and misunderstanding of the concept of small group teaching among students:

*“Large number of students in one group makes the students unable to take their chance during the class”.*

*“Students are not prepared well and they do not know their role in small group teaching”.*

*“Mentality of students because they are adapted to lecture and large group teaching till 4*^*th*^*year of study and the only thing they are aware about is how to pass the examination”.*

Most of the participants mentioned that the time allocated for each subject is short and not enough to cover all topics:

*“We have limited time to cover the subject because of holidays or any other problems. We lose many days in the course, so we have to cover all subjects in this short period of time”.*

One participant mentioned that the schedule of the course is not suitable:

*“The timetable of the lecture is not suitable. Students attend theoretical lectures after practical sessions. When they come to the hall, they are exhausted both physically and mentally”.*

The participants mentioned different challenges about assessment of students in small group teaching such as depending on knowledge and theory rather than practice and allocation of minimum marks on the interaction and daily activity of the students:

*“Most of the assessment methods are depending on the knowledge. Students feel that there no need for studying daily because they can pass examination easily if they only study for only one or two days before examination. The problem is in our strategy, which I think is not appropriate for small group teaching, because we are depending mainly in our assessment on knowledge of the students and not on the ethics and clinical skills of the students. I have seen students of the top ten graduates in the college but I think they are not ready to practice medicine in a safe way”.*

*“Assessment methods now focus more on theory rather than practical sessions”.*

Many administrative problems were reported by the participants such as time schedule of the lectures, hall allocation, lack of support staff and lack of monitoring of the implementation of this experience in different departments:

*“We do not have staff responsible for preparing halls and teaching facilities. Sometimes we have to wait for more than half an hour to find a room for teaching or sometimes we have to bring the projector from the college to the hospital”.*

### Students’ behavior in small group teaching

Most of the participants agreed that students’ behaviour during class is different from their behaviour in large group class:

*“Students are more active during small group teaching class and ask more questions because it is easier for them to ask in front of 30 students than 140–150 students. Students are more confident to ask and take part in the discussions”.*

Only one participant mentioned that he has noticed no difference between students’ behaviour in small group and large group:

*“I did not feel any difference. Students in both settings have no interest and do not like to participate in the classroom discussions”.*

Another participant mentioned that the situation is different between the two years of the study:

*“In the 5*^*th*^*year, there is very good discussion in the class and the students participate actively in the class; while in the 4*^*th*^*year, the students are passive and do not take part in classroom discussion especially the first group of students. This could be due to the fact that students do not have much information about the subject because they have not taken other subjects in detail”.*

Another participant mentioned that the behavior of the students in the class depends on the teacher and the teaching method:

*“It depends on the teacher and the teaching method. I gave some of my lectures as cases and there was good interaction”.*

### Teaching methods in small group teaching

Participants claimed that the main teaching method used in this new experience is still a traditional lecture method and depends mainly on the teacher but is more interactive:

*“The teaching method in the small groups is classical lecture method”.*

*“The main method is the traditional lecture method. Actually students do not have much role in the process of teaching in small groups”.*

### Teacher-student relationship in small group teaching

Participants agreed that this new way of teaching will improve teacher-student relationship:

*“I think small group teaching will improve and strengthen teacher-student relationship in a positive way because in the traditional curriculum there were barriers between students and teachers and students were shy and afraid to ask. Now the situation is changed but still there is mutual respect between the student and the teacher”.*

Participants emphasized that good relationship will enhance the learning process:

*“When the students see that the teachers have good relation with them, this will encourage them to study the subject”.*

*“If the teacher is happy, s/he will deliver more attractive lectures; and if the students are happy with their teachers, they will pay more attention to them. With good relationship the bridge between teacher and students will be removed and they feel more comfortable to ask questions and take part in the classroom discussions”.*

### Size of the small group

Participants stressed that the number of students in each group is still not ideal and should be less:

*“The number of students in each group is not ideal because an ideal small group should include 10–12 students”.*

One participant mentioned that there is difference between the number of the students in one group between the 4^th^ and the 5^th^ year of study:

*“Number of students in each group in the 4*^*th*^*year is large. While in 5th year, the number of students in each group is good”.*

Another participant mentioned that the task of the group will determine the size of the group:

*“The size of group depends on the task, for example, in theory 30 students is good but in clinical teaching the smaller number is more effective because medical teaching depends on personalization not like other humanitarian sciences. In small group teaching we do not have special limits. It depends on what we do, for example, if we have 150 students and divide them to groups with 30 students but if we have 300 students even 50 students in one group will be OK. The idea is how much we are able to make interaction with students and I think 30 students allow us to do that”.*

### Recommendations for improvement

Many recommendations have been made by the participants about different elements such as infrastructure, faculty members, students and curriculum. Recommendations about infrastructure included building more teaching halls suitable for small group teaching and providing adequate library with different resources:

*“There should be a very good infrastructure suitable for small group teaching like different small teaching rooms provided with different teaching facilities”.*

Participants argued that faculty members need more training and orientation about small group teaching:

*“Faculty members should receive more training about small group teaching and student centered learning approaches of learning. These approaches should be included in the program of teaching methods course in the university”.*

Three participants recommended increasing the number of faculty members in their departments:

*“Increasing the number of the faculty members in some departments”.*

Recommendations about students included decreasing the number of students in each group, more orientation of students about small group teaching, and involving them in the process of teaching:

*“Decreasing the number of students in each group”.*

*“More time should be spent with students for explaining small group teaching and how they should act in the session not just sitting or listening to what the teacher says. We have to teach them in a way that encourages them and makes them feel that this is a good method of teaching and is better than the other methods”.*

Six participants recommended modification and revision of the curriculum in different ways:

*“I think there is overload of the curriculum. Revision should be done for all the process”.*

*“I think the curriculum should be modified and focus more on the current issues, for example, now communicable disease is decreasing so we have to focus more on other subjects and the same thing should be done in other departments”.*

One participant recommended increasing the duration of the course:

*“I think there should be more time for the course or we have to give them another week at the end of the year for revision, for example, we can finish the course of medicine within seven weeks, so we can add the remaining week for revision at the end of the year”.*

Participants argued that the current teaching methods in small group should be changed to more interactive approaches:

*“The current teaching method should be changed (didactic method) because now most of the teachers are just reading from the slides”.*

Four participants recommended that only one examination should be done at the end of the course rather than two examinations:

*“Assessment methods should be changed, for example, now we are doing two main examinations, one at the end of the course and the other at the end of the year. It is better to eliminate the final examination of the year and to put all marks at the end of the course”.*

Six participants mentioned that the tools of assessment should be changed and focus more on daily activities of the students in the class and practical issues rather than knowledge:

*“I would like to see more focus on the practical examinations, although in some subjects we do not have practical examinations. Oral examination should be done at the end of each course because in oral examination we can asses students better”.*

*“Focusing more on the daily activities of the students in the class rather than the end of course, or mid and final year examinations”.*

Two participants recommended that small group teaching should be implemented from the first year of study in the college:

*“We have to start from the 1*^*st*^*year, because students will adapt to this type of teaching from the beginning”.*

Participants emphasized the need to improve the administration aspects such as time schedule, allocation of halls and the importance of monitoring and following up the implementation of small group teaching in different departments in the college:

*“It is better that the college monitors the implementation of the small group teaching among different departments because there is variation in implementation of this new method among different departments and faculty members”.*

## Discussion

This assessment was aimed to explore the perspectives of faculty members about the small group teaching experience implemented in both the 4^th^ and the 5^th^ year study in Hawler College of Medicine and to identify the main problems during implementation and opportunities for its improvement. The assessment process provides an opportunity to identify and address any area in which improvement may be made and to identify those aspects that reflect effective educational practice. Evaluation of any change or innovation in the study curriculum or methods of teaching from the faculty member’s perceptions is very vital for the educational environment. The current expansion in medical education renders evaluation of the effectiveness of our innovations together with the established modes of curriculum delivery very important. Moreover, qualitative data and communicating the results of evaluations with faculty and students are essential to successful reform [12].

### Positive aspects

Even though the experience of small group teaching in Hawler College of Medicine is relatively new, the participants were generally satisfied with the principles of small group teaching in medical education. They appraised its role in increasing the focus of students, encouraging student-teacher interaction, increasing students’ confidence, improving presentation skills of the students and building a better relation between teachers and students.

Small group teaching is an important component of undergraduate medical education; many medical schools around the world have adopted this strategy of teaching to make the classes more interactive and to give opportunities for students to take part in discussions [13,14].

Group discussion plays a valuable role in the education of students, allow students to express themselves and establish closer contact with academic staff. Discussion can also develop the more instrumental skills of listening, presenting ideas, persuading, and working as part of a team. Small groups can give students the chance to monitor their own learning and thus gain a degree of self direction and independence in their studies [5].

Several studies have reported advantages for small group teaching including increasing retention of knowledge, increasing opportunities to ask questions, enhancing learning to solve problems, enhancing transfer of concepts to new problems, increasing students’ interest, improving self-directed learning skills, enhancing ability to work as a team, increasing student-faculty and peer-peer interaction, improving communication skills, providing the opportunity to clarify points of confusion and offering opportunities for interactive demonstrations and students’ participation [15].

A meta-analysis carried out by Springer et al. on undergraduate small group teaching education since 1980 demonstrated that various forms of small-group learning are effective in promoting greater academic achievement and more favorable attitudes toward learning [16].

Walton carried out a review about small group in medical teaching and mentioned different advantages including increasing understanding of the subject, developing greater ability to present information, developing critical thinking, improving ability to ask questions, stimulating follow up of the subject further in private and independent study, increasing the ability to influence the content and methods of their work and obtaining instant feedback with each of the efforts they make [14].

Worral-Davies carried out a study to address ways in which learner can be actively involved in the small group setting. The author concluded that small group teaching provides an ideal opportunity for teachers to facilitate active learner participation [17].

Goshtasebi et al. carried out a study to compare students’ attitude and scores on small group teaching and lecture format among students enrolled in epidemiology course. The authors mentioned different advantages of small group teaching including active learning, application and development of ideas, improvement of deep learning, expansion of transferable skills such as leadership, encouragement of problem solving skills and improvement of time management [18].

Rathnakar et al. carried out a study to compare the effectiveness of large group lectures with small group teaching among the undergraduate students in the Department of Pharmacology, Kasturba Medical College, India. The authors mentioned that reducing the size of the class will produce many benefits for the teachers and the students. The students will receive more individual attention and the teachers will be able to manage the students better and spend less time for managing the students and more time can be utilized in teaching [19].

Other advantages have been mentioned by other authors such as familiarizing the students with an adult approach to learning, encouraging students to take responsibility for their own learning, promoting deeper understanding of material, encouraging problem-solving skills, developing communication and presentation skills, encouraging awareness of different views on issues, fostering active and collaborative learning and easing the distinctions between the better learner and the less efficient ones [4,20].

A cross-sectional study carried out by Aziz et al. to assess students’ perception of small group teaching among undergraduate students in Malaysia showed that small group teaching helps in the development of higher level intellectual skills such as reasoning and problem solving and the acquisition of interpersonal skills such as listening, speaking, arguing and group leadership [21].

It has also been reported that small group teaching has a direct positive effect on students’ motivation to learn, which has been shown to play a central role in promoting group productivity, elaboration of knowledge and interaction in different settings [22].

### Teacher-student relationship

Most of the participants emphasized the role of small group teaching in improving teacher-student relationship in the college. Many reasons were mentioned by the participants regarding this improvement including presence of a small number of students in each group and more interaction and contact between them.

During the past three decades, there has been an increase in research on the importance of teacher-student relationships. The quality of this relationship has been shown to be significantly associated with students’ social functioning, behavior problems, engagement in learning activities and academic achievement [23].

In efforts to better understand teacher-student relationship, some studies have focused on some of the characteristics of teacher-student relationship. Jacobson found that the first step in creating this type of environment is getting to know each student, thus providing the teacher with a better opportunity to develop a positive attitude that can in turn facilitate and support the students’ learning [24].

The influence of this relationship on learning could be weaker or stronger depending on the specific characteristics of the students and the teachers such as age, gender, ethnicity, socioeconomic status, learning difficulties of the students and teaching experience of the teachers [25].

### Problems and challenges

The participants reported a range of different challenges they have faced during implementation of this experience at Hawler College of Medicine such as lack of proper infrastructure, faculty members’ performance, problem related to the curriculum and administrative problems.

### Infrastructure

The participants particularly emphasized poor infrastructure and lack of appropriate teaching facilities for small group teaching. It has been reported in some other studies that the main challenges of small classes include the need to employ larger number of teachers and availability of adequate infrastructure with sufficient facilities and equipments [18,19]. This is of particular concern in developing countries and resource-constrained settings where financial allocations are limited. In fact, the building of Hawler College of Medicine was not designed to be a college; it was initially set to be a nursing school. Limited extension or renovation was done for the building to be more suitable for the college. Wars, civil strife and sanctions during the last few decades in Iraq and Kurdistan region have affected the development of medical education and availability of appropriate teaching facilities and resources [26-28].

Another problem regarding infrastructure raised by the participants was the lack of adequate library and poor access to internet which make the students struggling to find resources for their study. The call for more student-centered medical education would be, undoubtedly, much enforced with the inevitable use of information technology to support health care, life-long learning, education, research and management. Medical students should be able, at the time of graduation, to utilize biomedical information for solving problems, collecting, critiquing and analyzing information, taking action based on findings, communicating and documenting these processes [29].

### Faculty members’ performance

The participants were concerned about the teacher role in the process. Many themes regarding this subject were emerged from the interviews with faculty staff including the number of staff in some departments, training of the staff about new methods of teaching, resistance of the faculty members to the idea of small group teaching and commitment of the staff to the process of teaching in the college. Other studies have reported some of these problems including resistance from the faculty; the need to employ a larger number of teachers and the need for training the faculty members on working and managing small group teaching [8,18,19].

Regarding training of staff about small group teaching and student centered learning in general, Hawler Medical University in cooperation with University of Glasgow arranged a programme for training the staff about student centered learning approach. Thirty participants from Hawler Medical University were invited to enroll in this course and to take part in preparatory readings about student centered learning. The introductory reading was followed by four days of face to face academic development workshops for Hawler Medical University staff delivered by University of Glasgow staff in Erbil in March 2011. These workshops focused on the values and principles of student centered learning, adaptations to course design, teaching approaches and assessment, and observing and reflecting on teaching and learning practices. In September 2011, the University of Glasgow team returned to Erbil for a second visit and delivered three more days of the academic development program. The second visit has focused on the practical implementation and sustainability of student centered learning at Hawler Medical University. The University of Glasgow team designed three online activities for participants to complete between the two visits, which helped the participants to explore their understanding of student centered learning in the light of developing their knowledge of student centered learning [30]. However and as mentioned by some participants, the number of participants in this programme was limited and there was no follow up of the implementation of this approach by the participants in their classes.

The tutor is the backbone of the educational process. Despite many available educational strategies, all, with no exception, are tutor-dependent. This requires them to learn more about educational strategies and small group and individual learning, so that they become confident in delivering the curriculum successfully. Tutor development should be a continuous process not limited to the beginning of the implementation of new educational process [29].

Resistance of staff to this new approach could be attributed to the fact that most of them were adapted to the old style of teaching. Adopting a new approach needs more time and efforts from them. In the traditional educational systems, there is usually a form of resistance among teachers who use didactic teaching modalities to move towards a student centered system [19,29].

### Students

The faculty members reported some problems related to the students including unfamiliarity of students about small group teaching and their role in the process, inadequate orientation of students about new way of teaching, resistance from students to the new method and lack of students’ interest to the process.

Some of these concerns about the students in small group teaching have been reported in other studies including resistance from students, lack of interest on the part of participants in work in small groups, the students do not prepare for the sessions, one student dominates or blocks the discussion and the students want to be given the solutions to problems rather than discussing them [5,18,19]. In a study carried out by Rahman et al*.*, the authors have found in many cases that the students were not satisfied in working within a group and all members within a group did not participate equally in the discussions [31].

Lea et al. conducted a study on students’ attitudes to the student centered learning in a sample of 48 psychology students in the University of Plymouth. They found that, despite student centered policy in the university, 60% of the students had not heard the term [32].

The students’ resistance to small group teaching could be attributed to the fact that most of the students have only experienced the traditional method of teaching and are used to the teachers doing everything for them. Therefore, they might not be prepared to spend extra time on studying. Transitions between various teaching and learning styles should be subtle and gradual and requires good preparations and orientation of both students and teachers [21].

Regarding the size of each group, it was not possible to decrease the number of students in each group due to lack of infrastructure in the college. However, the number of students in a group in different universities is frequently fixed by curriculum demands and the total number of students in the university. The size of most groups in higher education may vary from a handful of students to around thirty [14,33].

Some authors emphasize that what characterizes a small group is not so much its size, but the teaching and learning context and the way in which the teacher works with and facilitates the learning process [34].

### Teaching in small groups

The study participants acknowledge that the Hawler College of Medicine has intended to implement more active learning methods but the actual teaching practice implemented is more accurately characterized as lectures with smaller groups of students holding the potential for more interaction. Research has recognized that teaching with smaller groups is more likely to facilitate student centered learning. The aim of small group sessions is to involve all the students in active discussion, and thereby facilitates active learning [4].

Learner centered teaching approaches are most effective in small groups and can be used to stimulate deep learning, and develop the students’ higher intellectual skills such as reasoning and problem solving [35].

The role of the teacher during transition from traditional teacher centred approach to more student centred approach should be changed. Many authors have emphasized on the role of the teacher in these new educational approaches. The shift from the traditional teacher centered approach, in which the emphasis is on teachers and what they teach, to a student centered approach, in which the emphasis is on students and what they learn, requires a fundamental change in the role of the teacher from that of a didactic teacher to that of a facilitator of learning [36].

Learner centered approaches challenge the traditional view of the teacher as the person who determines what, when, and how learners will learn, with didactic teaching as the predominant method. Creating an environment in which students can learn effectively and efficiently becomes the new prerequisite, demanding not only that teachers are experts in their fields but also and more importantly that they understand how people learn [37].

The University of Glasgow has identified four main strategies in a study on student centered learning practices in their University. The first strategy is to make the student more active in acquiring knowledge and skills. The second strategy is to make the students more aware of what they are doing and why they are doing it. The third strategy is a focus on interaction, such as the use of tutorials and other discussion groups. The final strategy is the focus on transferable skills [38].

### Assessment and examinations

The participants were concerned about the examination system and distribution of marks. Even though part of discussions was frequently going beyond the issue of small group teaching, proper setting of examination system and distribution of marks is essential for insuring fairness in any change in the teaching system. The competitive nature in the medical education and effect of marks on the future of students make the examination an important concern for them. The importance of the issue of examination and distribution of marks was also evident in the suggestions made to improve the system.

Assessment of students in new educational approaches has been emphasized by many authors. The use of the written summative examination is still a strong practice in today’s universities around the world [39]. Regarding short quizzes, which are frequently used as a tool of assessment in small group teaching, there are different opinions about it. Some of students feel that the quizzes are helpful in getting them to prepare for the small group session and in giving them feedback on their understanding of course content. Others feel that they are not helpful, and that they inhibit learning [40].

### Curriculum

Another important issue that was frequently mentioned by the participants was related to the curriculum. The participants complained about many issues such as curriculum overload, unclear syllabus with frequent changes in it without prior notification and the short duration of the course.

### Suggestions for improvement

The participants suggested many priorities to improve the current small group teaching experience in the college such as improving the infrastructure and teaching facilities, changing the teaching methods into more interactive way, changing the assessment system with focusing mainly on end of the course examinations rather than end year examinations and having clinical revisions before final year examinations, giving more active role to the students in designing the curriculum, decreasing the number of students in each group, proper orientation of students before implementing a new system, the necessity for providing training courses to teachers to be able to adopt to the new system effectively, implementing this method in early years of the study, better organization and management of the system and monitoring the implementation of this experience in different departments in the college.

Many of these suggestions have been reported in many other studies about small group teaching. Jacques mentioned that many steps should be done to encourage group interaction in small groups including a larger group should be broken into smaller groups of five or six students, membership should be organized on a heterogeneous or random basis to prevent cliques forming, skilled and sensitive handling of group process from within the group, imaginative management in the setting of tasks and organizing of purposeful activities for subgroups [5].

Dacre and Fox mentioned that it is often helpful to vary the activity of the small group by dividing the students into smaller subgroups, and ask them to consider a particular aspect of the topic, and then come together again after short period. It is also useful to stop at intervals to review the group’s progress [35].

Some authors have mentioned that more reduction in the size of the class may not improve the academic performance of the students in the group, if the teaching style continues to be as didactic lectures [19].

Involving students more actively in the teaching process is one of the principle elements in student centered learning approaches. In these approaches, the curriculum design allows for some choice within a programme of areas that students may study. It allows students to set some of their own learning objectives, dependent on prior knowledge, encourages the students to develop their own learning goals, thereby filling in the gaps in their knowledge or understanding [41].

Changing the role of the teacher in small group teaching has been emphasized by many authors. A good small group teacher facilitates communication between students, and between students and their teacher. To be good facilitators of communication, small group teachers need excellent questioning, listening, responding and explaining skills [17].

Orientation of students about small group teaching and their role is very important for successful teaching process. Reasons for the sessions and their purpose in the course should be clearly explained. Sometimes, it is necessary for the students to do some preparation, and this should be made clear in the beginning of the course [35].

Regarding assessment of students in small group teaching, many similar recommendations have been made by different authors. Assessment of students in small group teaching needs careful consideration and should be different from that of traditional methods and should include attendance, contribution, research, analysis, preparation of materials, support and encouragement of team members, practical contribution and end product [4].

The addition of more formative assessment, which emphasizes feedback to students on their learning, would enhance their learning. Examples of formative assessment include feedback on essays, written comments on assignments, grades during the year that do not add to end of year mark and multiple-choice questions for feedback only [39]. Ranking by formative assessment cannot be considered as the best evaluation tool of the lifetime-learner. Instead, achievement measured in the form of attainment of a particular number of competencies would, probably, be the future assessment tool [29].

Some studies have pointed out that, in practice, small group teaching is not always experienced as effective [26,42]. Regarding effective small group teaching, a study was carried out by Steinert to assess students’ perception of effective small group teaching in Medical College at McGill University in Canada. The findings of the study suggested that small groups should include effective small group tutors, a positive group atmosphere, active student participation and group interaction, adherence to small group goals, clinical relevance and integration cases that promote thinking and problem solving [40].

In another study carried out by Aziz et al. to assess undergraduate medical students’ perception about small group teaching at University Sains, Malaysia, the respondents identified that for a successful small group discussion all the participants must be mentally prepared to take part in active discussions and share knowledge and skills for in-depth understanding of the topic [21].

### Limitations of the study

Since this study has only involved Hawler College of Medicine, its findings might not be generalizable to other universities in Iraq because we cannot assume that the perspectives and attitudes expressed in one college apply to other contexts and other groups of faculty members and students. Such lack of generalizability does not mean that the findings of the study are not of value because the research illuminates how one group of staff is involved in grassroots implementation of reform and may generate theoretical insights that could be tested elsewhere. Such findings can help others consider their own situation and learn from the thoughts, feelings, and actions of the study participants. However, based on our knowledge we think that the general situation of the teaching methods in other universities in Iraq might be similar to our study to a large extent. Although this study characterizes the faculty responses as representing themes, they are, instead, direct responses to questions from the interview. This makes the study a reporting of responses rather than a pure qualitative analysis about what the participants have said. Finally, the operational definition of small groups in Hawler College of Medicine setting involved a much larger number of students than the ideal small groups in other settings. The large number of students in the study institution and the relatively large number of students included in the small groups add another limitation to this study as the findings do not represent the ideal size of small groups.

## Conclusions

Despite what the faculty perceived as the school’s failure to provide physical settings or training for small group learning to the faculty and the students, the faculty members were able to articulate positive experiences and outcomes associated with their school’s efforts to introduce teaching in smaller group sessions.

## Additional file

Additional file 1:
**Interview guide for assessing faculty perspectives of small group teaching experience in Iraq.**
