# Supplementary material for: A qualitative assessment of faculty perspectives of small group teaching experience in Iraq
Source: BMC Med Educ. 2015 Feb 15;15:19. doi: 10.1186/s12909-015-0304-7 (PMC4349223; doi:10.1186/s12909-015-0304-7)
Supplement: Additional file 1: — Interview guide for assessing faculty perspectives of small group teaching experience in Iraq. [file 12909_2015_304_MOESM1_ESM.pdf]

Additional file 1 - Interview guide for assessing faculty perspectives of small group teaching experience in Iraq

Q1- Was there preparation before implementing this new experience of small group teaching in the college? (Infrastructure, teaching staff and students)

Q2: What are positive aspects of the new small group teaching experience in the college?

Q3: What are problems & challenges of the new small group teaching experience in the college?

Q4: Do you think that there is any difference in student behavior in the small group and large group?

Q5: Size of small group?

Q 6: Teaching methods in small group teaching?

Q7. Do think that small group teaching affect teacher-student relationship?

If Yes, in which way?

Q 8: What are your recommendations for the future development of small group teaching in the college?
